# Determinants of health-related quality of life in patients with fracture of the axis vertebrae

**DOI:** 10.1038/s41598-021-98476-w

**Published:** 2021-09-24

**Authors:** Andzelina Wolan-Nieroda, Andrzej Maciejczak, Mariusz Drużbicki, Agnieszka Guzik

**Affiliations:** 1 Department of Physiotherapy, Institute of Health Sciences, College of Medical Sciences, University of Rzeszów, Rzeszow, Poland; 2 Department of Neurosurgery, St Luke Hospital, Lwowska 178 Street, 33-100 Tarnów, Poland

**Keywords:** Health care, Neurology

## Abstract

The study is designed to evaluate quality of life and functional performance in patients with type II and III odontoid fracture treated with anterior odontoid screw fixation. We investigated the relationship between quality of life and: (1) the range of axial rotation of the cervical spine, (2) neck pain intensity, and (3) level of disability in these patients. The study involved 60 patients operated on for type II and III fractures with the use of direct osteosynthesis of the dens. Quality of life and functional performance were assessed using SF-36 Questionnaire and Neck Disability Index (NDI). The range of axial rotation was examined with the use of the Zebris ultrasound system while the intensity of pain with the use of the VAS Visual Analog Pain Scale. The subjects’ quality of life was poorer with respect to the mental dimension (32.3%) compared to the physical dimension (22.7%). Based on the NDI survey, the rate representing the patients’ functioning in daily life amounted to 13.7% which reflects mild limitations in functional abilities. It was shown that the range of axial rotation (both to the right and the left) was not related to the degree of disability of patients as measured by the NDI questionnaire. The model of regression was statistically significant for overall quality of life (F = 48.24 *p* < 0.001), as well as physical dimension (F = 45.1 *p* < 0.001). Quality of life indicators in SF-36 are decreased in patients operated for type II and III odontoid fracture and the mental dimension of the quality of life is significantly poorer than the physical one. More than half of the patients operated for type II and III odontoid fracture regained normal functioning, as assessed with the NDI questionnaire.

## Introduction

The focus of sciences, including medical sciences, has shifted towards patients’ activity in various spheres of life, and consequently towards recognising their needs and expectations. The ability to function independently in domains related to the basic activities of daily living, both at home and outside, is a determinant of one’s quality of life. According to the World Health Organisation (WHO), quality of life reflects self-perceived physical and emotional condition, independence, self-reliance, and relation to the environment. A self-perceived quality of life is related to one’s level of physical and psychosocial functioning, assessed by reference to an ideal situation^1–3^. The level of the quality of life provides information about subjects’ perception of their health status^4^. Many patients with spinal diseases emphasise that good quality of life for them is more important than the duration of life. Therefore, assessment of the quality of life should be an obligatory component of decision making on the treatment applied to the patient in the changing life situation^4–7^.

Odontoid fractures of the axis require operative treatment in majority of cases. Following an injury, the majority of patients do not present neurological symptoms. Treatment of such fractures is aimed on gaining fusion through either conservative management, namely immobilization in hard collar or surgery. Surgical treatment may result in severe reduction of the range of motion of the upper cervical spine in all planes. This translates into decrease in range of motion of head against torso. For example, flexion between atlas and axis constitutes nearly one fourth of total range of head flexion against the torso. The same refers to the range of head extension. However, the most prominent of all motions at C1/C2 segment is axial rotation which reaches 45 degrees towards one side. It provides nearly half of total range of axial rotation of head against torso^8–10^. That is why we included this parameter into our analysis. Axial rotation can be severely compromised following surgical treatment. Depending on the surgical method applied, the range of axial rotation of head may be reduced by half or even more. This occurs when a surgical treatment involves fusion between C1 and C2 or even between occiput and the upper or midcervical spine. Fortunately, majority of odontoid fractures is treatable with direct osteosynthesis of the axis peg with the use of lag screw which is theoretically a motion sparing technique. Direct osteosynthesis of the odontoid peg theoretically leaves the motion between atlas and axis unaltered. Surprisingly this technique, according to our previous observations, may not prevent decrease in the range of atlanto-axial motion^8^. Furthermore, decreased mobility at the upper cervical spine co-occurs with continuous pain^9^. Given the above, chronic body pain may lead to a sense of disability and to decreased quality of life in these patients. Therefore, the problem should be investigated.

Based on literature review it can be concluded that the present study is the first to assess quality of life in relation to such factors as the range of cervical axial rotation, pain and disability and to investigate their adverse effects in self-assessed life satisfaction of patients following axis fracture. Motivation for the current study was inspired by our earlier research findings, which showed that subjects treated surgically for odontoid fractures present statistically significant lower ranges of active motion of the cervical spine, particularly in axial rotation than the healthy population^8–10^.

The study is designed to evaluate quality of life and functional performance in patients with axis fracture and to assess the relationship between quality of life and the range of cervical spine rotation, pain severity, and level of disability in patients, following this type of injury.

## Material and methods

### Participants and setting

The study involved a consecutive series of patients operated on for type II and III odontoid fracture in a neurosurgical ward of a county hospital between 2016 and 2017. The study group included only patients operated on with the use of anterior odontoid screw, at least 2 years post surgery, with complete bony fusion of the fracture as confirmed by CT or by dynamic X-rays. The study group comprised of 60 subjects (18 females and 42 males), with the mean age of 49.2 ± 18.3 years (ranging from 18 to 80).

### Study protocol

All the subjects received detailed information about the purpose and procedure of the study and agreed to participate in writing. Informed consent was given by the all participants. Approval to conduct this cross-sectional study was obtained from the Bioethics Commission at the University of Rzeszow, Poland, and all the methods were performed in accordance with the relevant guidelines and regulations.

### Procedures and data analyses

Measurements of range of motion were performed in a spine kinesiology laboratory of University of Rzeszow, while questionnaires were filled in by patients of neurosurgical ward at the county hospital on the occasion of their check up. Information collected about each patient comprised personal data, medical history of the illness, including diagnoses specified in the available records, such as CT and X-ray reports, as well as limitations in everyday activities. The Zebris System with cervical spine software was used for the study^11^. The angular measurement of the range of motion expressed in degrees, concerned rotation movements. The range of motion was measured in the transverse plane, in a sitting position with the back support. Ranges of motion in the study group were compared to the ranges of motion of the cervical spine according to ISOM (International Standard Orthopedic Measurements)^12^ norms.

The intensity of pain was assessed with the use the VAS Visual Pain Scale^13^.

The global assessment of functional performance and quality of life was performed using standardised questionnaires. These included Neck Disability Index (NDI) survey, designed for patients with neck pain. The tool measures the quality of physical performance, and consequently the quality of life, as subjectively assessed by the patient. It comprises questions related to ten aspects of daily functioning, divided into the following sections: pain intensity, personal care, lifting, reading, headaches, concentration, work, driving, sleeping and recreation. In each section there are six possible answers, rated from 0 to 5. Responses to the questions enable assessment of the patient’s functioning in everyday activities and are classified on a scale from 0 to 5. The total score in all the sections of the questionnaire ranges from 0 to 50 points. The results may also be presented as per cent values, from 0% (0 points) to 100% (50 points). Higher score reflects poorer quality of life and greater physical dysfunction. Values in the range of 0–19% reflect a lack of disability, 20–39%—mild disability, 40–59%—moderate disability, 60–79%—severe disability, and the values in the range of 80–100% correspond to full disability, i.e. limited motor functions resulting in a need to remain in a lying position due to pain^14,15^.

Assessment of the patients’ quality of life was performed using SF-36 Survey, consisting of 36 questions which allow to evaluate mental and physical health. Some questions require a yes/no answer, in others responses are rated from 1 to 5. A total of 171 points can be scored. A higher score corresponds to poorer functional status in the specific spheres, and consequently reflects lower overall quality of life. The SF-36 Survey addresses the following eight domains: physical functioning, physical role limitations, bodily pain, general health perceptions, vitality, social functioning, emotional role limitations and, mental health. Specific domains are combined into two SF-36 quality-of-life dimensions: physical dimension and mental dimension. Subsequently the two quality-of-life dimensions are added (physical dimension + mental dimension = TOTAL SF-36), to obtain the final indicator of the patient’s quality of life according to SF-36 scale^16–18^.

Statistical analyses of the collected material were computed using Statistica 10.0 software. Consistency of the distributions with the normal distribution was verified with Shapiro–Wilk W test and homogeneity of variance was assessed with Levine’s test. Basic descriptive statistics calculated included: mean, median, minimum and maximum values as well as standard deviation. Spearman's rank correlation coefficient was used to assess the correlation of cervical range of motion to the quality of life measured using SF-36 scale and to disability level measured by NDI survey, and to examine relationships between the subjects’ scores achieved in all the scales, i.e. VAS, NDI and SF-36. Multiple regression analysis was applied in order to obtain quantitative presentation of the associations between multiple independent variables (cervical range of motion [Zebris], pain severity [VAS] and disability level [NDI]) and the dependent variable (SF-36 quality of life) and to show the simultaneous effect of the four factors. Statistical significance was assumed if *p* < 0.05.

### Sample size calculation

The sample selection calculator was used to calculate the minimum sample size, taking into account the total number of patients with odontoid fracture treated yearly in the ward. A fraction size of 0.7 was applied, with a maximum error of 5%, and as a result, a sample size of 51 patients was established.

## Results

### Range of cervical spine rotation

The Zebris ultrasound system showed the following range of cervical spine rotation in study group: rotation to the left 40.6 + 19.3.; rotation to the right 41.7 + 20.0. The values of range of cervical spine mobility of the study group were then compared to the ISOM norms (rotation to the right and left respectivly 50). The obtained results were highly statistically significantly different from the accepted ISOM norms (Student’s t-test; *p* < 0.0001***). The ranges of mobility of the cervical spine of patients in the study group for both right and left rotation were significantly limited in relation to the ISOM norms—Table 1.

**Table 1:** Mobility range of cervical spine in study group and its comparison with ISOM norms.

Mobility range [Zebris]	Study group N = 60					Norma ISOM	*P*	
	$\overline{x}$	Me	Min	Max	S		t	*P*
Rotation to the left	40.6	39.0	5.0	90.0	19.3	50	− 4.34	0.0000
Rotation to the right	41.7	40.0	5.0	90.0	20.0	50	− 3.72	0.0000

### Quality of life and functional performance

Analysis of the subjects’ quality of life took into account their total scores acquired in SF-36, and their results in the two quality-of-life dimensions: physical and mental. It was shown that patients experienced the least restrictions in performing roles due to physical health (9.5%). There was also a low indicator indicating a small problem related to their emotional functioning (21.1.8%). High indicators on the SF-36 scale were obtained for aspects related to the mental dimension—general health (49.5%), vitality (41%), mental health (34.6%) and pain (28.9%). The quality of life of the respondents turned out to be worse in the mental dimension (32.3%) than in the physical dimension (22.7%). The overall quality of life in the entire SF-36 questionnaire was rated at an average of 26.5% (Table 2).

**Table 2:** Characteristics of the subjects’ quality of life, assessed with SF-36 questionnaire.

SF-36	Study group N = 60					Max possible score	Percent score (%)
	$\overline{x}$	Me	Min	Max	S		
Physical functioning	4.75	0.00	0.00	50.00	10.27	50	9.5
Physical role limitations	4.17	0.00	0.00	20.00	7.20	20	20.8
Bodily pain	2.60	2.00	0.00	7.00	2.26	9	28.9
General health perceptions	11.88	12.00	5.00	18.00	2.54	24	49.5
Vitality	8.20	8.00	4.00	15.00	2.33	20	41.0
Social functioning	1.95	2.00	0.00	5.00	1.59	8	24.4
Emotional role limitations	3.17	0.00	0.00	15.00	5.52	15	21.1
Mental health	8.65	9.00	3.00	15.00	2.94	25	34.6
Physical dimension	23.40	14.50	5.00	79.00	18.67	103	22.7
Mental dimension	21.97	20.00	8.00	50.00	10.09	68	32.3
Overall SF-36 quality of life	45.37	34.00	15.00	115.00	27.38	171	26.5

$\overline{x}$ mean, *Me* median, *S* standard deviation, *Min* minimum value, *Max* maximum value.

Analysis of the patients’ responses to the NDI survey related to their functioning in everyday life, showed that the subjects most frequently complained about cervical pain and about inability to lift weights. The patients experienced difficulties performing work or driving, and reported headaches. On average the subjects scored approximately 6.8 points, out of the 50 points possible. The mean rate representing the quality of the patients’ functioning in daily life amounted to 13.7% which reflects mild limitations in functional capacities. The mean scores in the specific sections of the NDI survey are shown in Table 3*.*

**Table 3:** Mean scores in the sections of NDI questionnaire.

NDI	Study group N = 60				
	$\overline{x}$	Me	Min	Max	S
Pain intensity	1.2	1.0	0.0	4.0	1.2
Personal care	0.6	0.0	0.0	3.0	0.7
Lifting	1.1	1.0	0.0	4.0	1.2
Reading	0.5	0.0	0.0	4.0	0.9
Headaches	0.8	1.0	0.0	4.0	0.7
Concentration	0.3	0.0	0.0	3.0	0.6
Work	0.8	0.0	0.0	5.0	1.3
Driving	0.8	1.0	0.0	5.0	1.2
Sleeping	0.4	0.0	0.0	3.0	0.6
Recreation	0.3	0.0	0.0	3.0	0.7
NDI—total score	6.7	5.0	0.0	30.0	6.8
NDI—per cent score	13.5%	10.0%	0.0%	60.0%	13.7%

$\overline{x}$ mean, *Me* median, *S* standard deviation, *Min* minimum value, *Max* maximum value.

Interpretation of the NDI scores shows normal quality of life in 78.3% of the subjects, mild disability in 16.7% and only 5.0% moderate disability (Table 4).

**Table 4:** The subjects’ disability level according to NDI.

Disability level according to NDI	Study group	
	N	%
No disability (up to 20%)	47	78.3
Mild disability (21–40%)	10	16.7
Moderate disability (41–60%)	3	5.0
Severe disability (61–80%)	0	0.0
Total disability (81–100%)	0	0.0
Total	60	100.0

Analysis of the relation between the subjects’ range of cervical spine rotation and disability level showed that the range of cervical spine rotation (both right and left) in Zebris measurement was related to the patients’ disability level, measured by the NDI questionnaire (R = − 0.4, *p* = 0.0001). The greater cervical range of motion the lower level of disability. In the case of the SF-36 questionnaire a weak correlation was only identified in the case of left rotation (R = − 0.3, *p* = 0.0019).

### Multiple regression analysis

The obtained regression model explained approximately 57% of the variation in the overall quality of life (R^2^ = 0.57, adjusted R^2^ = 0.54). The regression model is statistically significant (F = 18.32 *p* < 0.001). Statistically significant factors were found in the case of the variables: VAS (*p* = 0.010) and NDI (*p* < 0.001). This means that the variables are significantly related to the total score in SF-36. The score of SF-36 (overall quality of life) is most visibly linked to NDI (R = 0.56). The results of the measurements carried out with the use of the VAS and NDI were positively correlated to quality of life—the greater limitations identified by NDI and the higher pain intensity, the greater limitations identified by SF-36, i.e. poorer quality of life. There was not a correlation between quality of life and results of measurements assessing right and left neck rotation. Based on the value of “b” parameter it can be concluded that a 1% increase in NDI-related limitations leads to a decline in the quality of life by 1.23%. An one-point increase in pain intensity in VAS leads to a decline in the quality of life by 2.16%—Table 5.

**Table 5:** Assessment of overall quality of life depending on range of rotation, pain intensity and disability level (Zebris / VAS / NDI).

SF-36 Quality of life (%)	Multiple regression						
	R^2^	Adjustd R^2^	F	Regressnmodel *p*	b	Partialcorrelation	*P*
Zebris right rotation	0.57	0.54	18.32	*p* < 0.001	− 0.02	− 0.02	0.889
Zebris left rotation					− 0.09	− 0.07	0.580
VAS					2.16	0.34	0.010
NDI					1.23	0.56	< 0.001

R^2^, regression model; Adjusted R^2^, regression model, removes extreme values; F, Fisher’s test result; b, regression coefficient; partial correlation, the variable Xi is correlated with the variable Y after accounting for effects of all the remaining independent variables (simultaneous effect of all the four factors); *p* significance level.

The obtained regression model explained approximately 55% of the variation in the quality of life in physical dimension (R^2^ = 0.55, adjusted R^2^ = 0.52). The regression model is statistically significant (F = 16.83 *p* < 0.001). Statistically significant factors were found in the case of the variables: VAS (*p* = 0.004) and NDI (*p* < 0.001). It means that the variables are significantly related to the SF-36 score in physical dimension. The SF-36 score related to physical dimension is most visibly linked to NDI (R = 0.50). The results of the VAS and NDI measurements were positively correlated to quality of life. There was not a correlation between quality of life and results of measurements assessing right and left neck rotation. Based on the value of “b” parameter, it can be concluded that a 1% increase in NDI-related limitations leads to a decline in the quality of life by 1.22%. An one-point increase in pain intensity in VAS leads to a decline in the quality of life by 2.86%—Table 6.

**Table 6:** Assessment of physical dimension quality of life relative to range of rotation, pain intensity and disability level (Zebris / VAS / NDI).

SF-36 Quality of life (%)	Multiple regression						
	R^2^	AdjustedR^2^	F	Regressmodel *p*	b	Partialcorrelation	*P*
Zebris right rotation	0.55	0.52	16.83	*p* < 0.001	− 0.01	− 0.01	0.954
Zebris left rotation					− 0.14	− 0.10	0.479
VAS					2.86	0.38	0.004
NDI					1.22	0.50	< 0.001

R^2^, regression model; Adjusted R^2^, regression model, removes extreme values; F, Fisher’s test result; b, regression coefficient; partial correlation, the variable Xi is correlated with the variable Y after accounting for effects of all the remaining independent variables (simultaneous effect of all the four factors); *p*, significance level.

The obtained regression model explained approximately 48% of the variation in the quality of life in mental dimension (R^2^ = 0.48, adjusted R^2^ = 0.44). The regression model is statistically significant (F = 12.52 *p* < 0.001). Statistically significant factors were found in onlly the case NDI (*p* < 0.001). It means that the variables are significantly related to the SF-36 score in mental dimension. The score of SF-36 is most visibly linked to NDI (R = 0.56). The results of the NDI measurements were positively correlated to quality of life. Based on the value of “b” parameter it can be concluded that a 1% increase in NDI-related limitations leads to a decline in the quality of life by 1.24%—Table 7.

**Table 7:** Assessment of mental dimension quality of life depending on range of rotation, pain intensity and disability level (Zebris / VAS / NDI).

SF-36 Quality of life (%)	Multiple regression						
	R^2^	Adjusted R^2^	F	Regressionmodel *p*	B	Partial correlation	*P*
Zebris right rotation	0.48	0.44	12.52	*p* < 0.001	− 0.04	− 0.03	0.806
Zebris left rotation					− 0.02	− 0.02	0.887
VAS					1.11	0.18	0.188
NDI					1.24	0.56	< 0.001

R^2^, regression model; Adjusted R^2^, regression model, removes extreme values; F, Fisher’s test result; b, regression coefficient; partial correlation, the variable Xi is correlated with the variable Y after accounting for effects of all the remaining independent variables (simultaneous effect of all the four factors); *p*, significance level.

## Discussion

Our literature search yielded no studies investigating quality of life in patients following axis fracture. The current study seems to be the first one which, in addition to assessing the quality of life and disability level, examines the associations between quality of life and the range of cervical spine rotation, pain intensity and disability level in patients following axis fracture.

The present study showed that rotation range of motion in cervical spine in the study group was highly statistically significantly limited in comparison to ISOM norms. Those results comply with our previous study showing that subjects treated surgically for odontoid fractures present statistically significant lower ranges of active motion of the cervical spine, particularly in axial rotation than the control group (*p* < 0.001)^8,10^.

The findings show that the patients experienced problems due to physical and emotional health. On the other hand high scores of the SF-36 scale were observed in the domains associated with mental dimension, i.e. general health perceptions, vitality, social functioning, and pain. The subjects’ quality of life was poorer with respect to the mental dimension compared to the physical dimension. On average the overall SF-36 quality of life was assessed at the level of 26.5%. A study by Poznańska focusing on the quality of life in patients with a history of spinal pathology showed that the subjects’ quality-of-life related perceptions were differentiated by the type of the disorder. The highest quality of life was identified in the case of oncological patients (30%), and the lowest in patients with post-traumatic fractures (24%)^19^.

The present study also investigated a relationship between the range of cervical spine axial rotation and the patients’ self-perceived quality of life. It was shown that both left and right rotation is not significantly correlated with the patients’ disability level as assessed by the NDI and SF-36 questionnaire. Riddle and Stratford reported a positive correlation between axial neck rotation and MH domain in the SF-36 questionnaire^20^. Conversely, Hermann and Reese did not observe associations between the mobility of the upper section of the spine and the psychological assessment in the SF-36 questionnaire among patients with neck pain syndromes^21^. Guzy et al. showed a negative relation of trait and state anxiety to the movement of head protraction in patients with neck pain syndromes. They did not find associations between spine mobility and other negative emotions. The authors emphasise a need for further study of links between spine mobility and emotional states, since in the literature there are very few reports focusing on relationships between mobility and psychological factors^22^.

The present study shows that results in NDI scale and VAS are significantly related to SF-36 scores. Previous research findings based on the SF-36 questionnaire, among the patients experiencing pain in the cervical region, showed poorer quality of life. Daffner et al. reported that SF-36 scores reflected lower vitality and poorer general health perceptions in patients with symptoms located in the cervical spine compared to the subjects with symptoms occurring in the upper limbs^5^. It means that pain is associated with the general self-perceived quality of life in individuals experiencing pain in the cervical spine.

The present study shows that in study group the mental dimension of the quality of life is significantly poorer than the physical dimension. Sutton, et al. suggest that depression and negative emotions are significantly correlated to the self-perceived quality of life^6^. Furthermore, Szczygieł et al. demonstrated that negative emotions, such as worry, fear and anxiety, correlate positively with the duration of neck pain^23^. Likewise, Guzy et al. observed that state anxiety and negative emotions are positively associated with the intensity of neck pain^22^.

Odontoid fractures seems to affect person’s functioning in daily life, which results in poorer standard of living and lower sense of security. This is due to the fact that the injury leads to decrease in cervical spine axial rotation, accompanied with persisting pain experienced during many basic activities of everyday life. Consequently, following such an injury some individuals negatively assess their functional capacities, and that adversely affects their self-perceived quality of life. Furthermore, in accordance with the present findings the mental dimension of the quality of life in patients with axis fracture, appears to be far poorer than the physical dimension. Given the above, multidisciplinary approach should be applied in treatment of axial skeleton injuries and the patients should receive psychological support as soon as possible.

### Study limitations

The results of the present study concerns patients with odontoid fracture treated with anterior odontoid screw fixation. On the one hand the strong suit of this study is strictly selected study group with one type of performed surgery. On the other hand, it is necessary to continue research and compare the results with odontoid fracture patients treated with different type of surgery.

The presented results should be treated as preliminary ones and should be followed by a study involving a larger group. However, despite the small number of participants, the sample size calculation proved that our sample of participants was sufficient for the feasibility study.

Further research should also include the other factors influencing the quality of life of patients such as gait analyses and severity of pain in cervical spine after the fracture.

## Conclusion

Quality of life indicators in SF-36 are decreased in patients operated for type II and III odontoid fracture while the mental dimension of the quality of life is significantly poorer than the physical one. More than half of the patients operated for type II and III odontoid fracture regained normal functioning, as assessed with the NDI questionnaire.

## Abbreviations

SF: Quality of life questionnaire
NDI: Neck disability index
VAS: Visual analog pain scale
